# Anlotinib plus chemotherapy for T790M‐negative *EGFR* ‐mutant non‐sqNSCLC resistant to TKIs: A multicenter phase 1b/2 trial

**DOI:** 10.1111/1759-7714.14713

**Published:** 2022-11-08

**Authors:** Juan Li, Yuke Tian, Min Zheng, Jun Ge, Jiliang Zhang, Dejun Kong, Mei Chen, Ping Yu

**Affiliations:** ^1^ Department of Medical Oncology Sichuan Cancer Hospital & Institute, Sichuan Cancer Center, School of Medicine, University of Electronic Science and Technology of China Chengdu China; ^2^ Department of Oncology Chengdu Seventh People's Hospital, Chengdu Tumor Hospital Chengdu China; ^3^ Department of Oncology The Second Affiliated Hospital of Chengdu College, Nuclear Industry 416 Hospital Chengdu China; ^4^ Department of Respiratory Medicine Chengdu Fifth People's Hospital Chengdu China

**Keywords:** anlotinib, chemotherapy, epidermal growth factor receptor, non‐small cell lung cancer, T790M

## Abstract

**Background:**

This multicenter phase 1b/2 trial aimed to explore the maximum tolerated dose (MTD), activity, and safety of anlotinib plus chemotherapy in patients with T790M‐negative epidermal growth factor receptor (*EGFR*)‐mutant advanced nonsquamous non‐small cell lung cancer (NSCLC) after resistance to first‐ or second‐generation EGFR tyrosine kinase inhibitors (TKIs).

**Methods:**

In the phase 1b stage, patients received anlotinib (8/10/12 mg, days 1–14) combined with cisplatin (75 mg/m^2^, day 1) or carboplatin (AUC = 5, day 1) plus pemetrexed (500 mg/m^2^, day 1) for a 3‐week cycle based on a 3 + 3 dose‐escalation design. In the phase 2 single‐arm stage, anlotinib was administered at MTD combined with platinum plus pemetrexed for four cycles, followed by anlotinib maintenance therapy. The primary endpoint of the phase 2 stage was progression‐free survival (PFS).

**Results:**

The study was prematurely terminated due to slow accrual after 19 patients had been enrolled between January 18, 2019, and March 21, 2021. The MTD of anlotinib was 12 mg. The median PFS was 5.75 (95% confidence interval, 4.37–7.52) months. The objective response rate was 47.4% (95% confidence interval, 24.5%–71.1%). In the 12 mg group, seven (58.3%) patients experienced grade 3–4 treatment‐related adverse events, and the most common ones were hypertension (6 [50.0%]), decreased platelet count (2 [16.7%]), and hypertriglyceridemia (1 [8.3%]). No treatment‐related deaths occurred.

**Conclusion:**

Anlotinib plus platinum and pemetrexed showed promising antitumor activity with manageable toxicity in patients with T790M‐negative *EGFR*‐mutant advanced nonsquamous NSCLC after progression on first‐ or second‐generation EGFR TKIs.

## Graphical abstract

Patients with T790M‐negative *EGFR*‐mutant advanced NSCLC received anlotinib plus platinum‐based chemotherapy after resistance to first‐ or second‐generation EGFR TKIs. The maximum tolerated dose (MTD) of anlotinib was 12 mg, and the median PFS was 5.75 (95% CI: 4.37–7.52) months. This combination therapy might be a potential alternative option as a second‐line treatment.

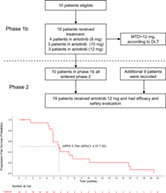

## INTRODUCTION

Lung cancer is the second most common cancer and leads to almost a quarter of all cancer deaths worldwide. There are an estimated 2.2 million new cases, and 1.8 million deaths in 2020.^1^ Non‐small cell lung cancer (NSCLC) accounts for approximately 85% of lung cancer, and many patients already have locally advanced or metastatic NSCLC at initial diagnosis.^2^ Targeted therapy is normally adopted based on mutation status for patients with advanced NSCLC. Epidermal growth factor receptor (*EGFR*) tyrosine kinase inhibitors (TKIs) are considered the first‐line therapy for patients with advanced NSCLC harboring activating *EGFR* mutation.^3^ Nevertheless, first‐ or second‐generation TKIs become resistant after a median progression‐free survival (mPFS) of 10–14 months. About 50%–60% of patients commonly have T790M mutation as acquired resistance.^4^ A third‐generation EGFR TKI osimertinib was approved for patients with *EGFR* T790M‐positive metastatic NSCLC progressing after the failure of first‐ or second‐generation EGFR TKIs,^5^ but treatments for those with T790M‐negative acquired resistance are extremely limited.

Platinum‐based doublet chemotherapy, which is used for patients with *EGFR*‐negative advanced NSCLC, is currently adopted for patients with T790M‐negative *EGFR*‐mutant advanced NSCLC,^3^ but the prognosis remains unsatisfactory. A numerically shorter overall survival (OS) was found with the addition of gefitinib to cisplatin plus pemetrexed (21.4 vs. 22.5 months) in the T790M‐negative subgroup among patients with *EGFR*‐mutant NSCLC after progression following the first line gefitinib therapy in the IMPRESS study.^6^ A single‐arm phase 2 trial (CT18) evaluated the antitumor activity of toripalimab, an antiprogrammed cell death‐1 antibody, plus carboplatin and pemetrexed in a similar setting, and the results showed a limited mPFS of 7.0 months.^7^ Based on the aforementioned data, it is still necessary to explore new effective regimens.

Anlotinib, a multitarget antiangiogenic agent, has been approved as a third‐line drug for advanced NSCLC in China.^8^ Anlotinib reversed acquired resistance to EGFR TKIs via fibroblast growth factor receptor 1 (FGFR1) signaling pathway in T790M‐negative *EGFR*‐mutant NSCLC in an in vitro trial.^9^ Several studies identified the feasibility of anlotinib plus chemotherapy in the treatment of advanced NSCLC.^10^, ^11^ The combination of antiangiogenic agent and chemotherapy may be a novel development direction for T790M‐negative *EGFR*‐mutant advanced NSCLC.

Therefore, this study aimed to explore the maximum tolerated dose (MTD), activity, and safety of anlotinib combined with platinum plus pemetrexed in patients with T790M‐negative *EGFR*‐mutant advanced nonsquamous NSCLC (non‐sqNSCLC) after acquiring resistance to first‐ or second‐generation EGFR TKIs.

## METHODS

### Study design and participants

This was a single‐arm phase 1b/2 trial that was carried out in four clinical centers (Table S1) in mainland China from January 18, 2019, to March 21, 2021.

The main inclusion criteria were (1) age of 18–75 years; (2) histologically or cytologically confirmed stage IV non‐sqNSCLC; (3) harbored *EGFR* mutation by gene sensitive mutation test; (4) prior treatment of first‐ or second‐generation EGFR TKIs and progression after complete response (CR) or partial response (PR) for over 4 months, or stable disease (SD) for at least 6 months according to the Response Evaluation Criteria In Solid Tumors (RECIST) version 1.1,^12^ and confirmed negative T790M by droplet‐digital polymerase chain reaction, amplification refractory mutation system, or next‐generation sequencing; (5) Eastern Cooperative Oncology Group (ECOG) performance status of 0 or 1; (6) life expectancy of over 3 months; and (7) adequate hematological, renal, and hepatic functions.

The primary exclusion criteria were: (1) combined with small‐cell lung cancer or non‐small cell adenosquamous carcinoma with squamous cell carcinoma as the dominant component; (2) clinically significant hemoptysis (>50 ml every day) within 3 months, other clinically significant bleeding symptoms, or evident bleeding tendency; (3) uncontrolled symptoms of brain metastases; (4) tumor with ≤5 mm distance from a great blood vessel, a central‐type tumor that invaded great blood vessel with ≤2 mm distance from the bronchial tree, tumor with an evident cavity, or necrotic tumor; (5) uncontrolled hypertension; (6) coagulation disorders, with a bleeding tendency or thrombolytic therapy or anticoagulation therapy; (7) arterial/venous events within 12 months, such as a transient ischemic attack, cerebral hemorrhage, cerebral infarction, deep venous thrombosis, and pulmonary embolism; or (8) other situations judged by the investigators, which would have prompted patients to discontinue participation in the study.

This study was approved by the ethics committees of all participating centers, and all patients provided written informed consent. The study was registered with ClinicalTrial.gov (NCT03706287).

### Procedures

For the dose escalation part of this trial in the phase 1b stage, a standard 3 + 3 design was employed. Three doses of anlotinib, 8, 10, and 12 mg, were administered with 8 mg as the initial dose. On days 1–14, anlotinib was administered, and cisplatin at 75 mg/m^2^ or carboplatin at an area under the curve (AUC) of 5 mg/ml/min plus pemetrexed 500 mg/m^2^ were administered on day 1 of a 3‐week cycle. The study would be terminated if dose‐limiting toxicity (DLT) occurred in two patients in the 8 mg group. MTD was defined as the highest dosage when DLT appeared in <33% of the patients. If DLT occurred in 33% or more of patients in the 10 or 12 mg groups, then the resultant dose was considered MTD.

DLT was defined as an treatment‐related adverse event that met the following criteria: grade 3 febrile neutropenia; grade 3 neutropenia lasting for >7 days; clinically significant bleeding or signs requiring platelet infusion; grade 3 thrombocytopenia that lasts for >7 days or grade 4 thrombocytopenia; grade 4 anemia; grade 2 hyperbilirubinemia that lasts for >7 days or grade 3 hyperbilirubinemia; grade 3 increased alanine transaminase (ALT)/aspartate transaminase (AST) lasting for >7 days or grade 4 increased AST/ALT; grade 2 pancreatitis; grade 3 asymptomatic elevated amylase and/or asymptomatic elevated lipase that did not resolve to grade 2 within 7 days, which was judged attributable to pancreas injuries; corrected QT interval by Fridericia's formula >500 ms, or other grade ≥3 cardiac toxicities; grade 2 hand‐foot syndrome (HFS) lasting for >7 days, or grade ≥3 HFS; grade 2 hypertension lasting for >7 days, or grade ≥3 hypertension; any grade of cerebral hemorrhage, grade >2 pulmonary hemorrhage, grade >3 other hemorrhage, or grade 2 hemorrhage lasting for >7 days; grade 2 coagulation abnormality that lasts for >7 days; grade 2 proteinuria that lasts for >7 days or grade ≥3 proteinuria; grade 3 fatigue lasting for >7 days or grade 4 fatigue; and any other grade ≥3 nonhematological toxicities that did not alleviate after symptomatic treatment, or grade 2 nonhematological toxicities lasting for >14 days after symptomatic treatment.

In the phase 2, the dose expansion part of this trial, anlotinib was administered at MTD on days 1–14, and cisplatin 75 mg/m^2^ or carboplatin (AUC = 5) plus pemetrexed 500 mg/m^2^ were administered on day 1 of a 3‐week cycle for four cycles. MTD of anlotinib as maintenance therapy (14 days, followed by a 7‐day break) was continued until disease progression, intolerable toxicity, or withdrawal of consent. When grade 3 hematological toxicities or grade 2 nonhematological toxicities occurred, the dose was reduced or interrupted. The cumulative interruption time in each treatment cycle should be maintained at <14 days, and the treatment interruption should be kept less than twice in each cycle.

Imaging examinations by computed tomography (CT) or magnetic resonance imaging (MRI) were employed at baseline and every 6 weeks. The response was assessed by the investigator based on RECIST 1.1.^12^ Adverse events (AEs) were recorded during the study period and graded according to National Cancer Institute Common Terminology Criteria for Adverse Events (CTCAE) version 4.0.3.

### Outcomes

The primary endpoint of the phase 2 part was PFS, defined as the time from the start of treatment to disease progression or death from any cause. Secondary endpoints were objective response rate (ORR, defined as the proportion of patients with the best overall response of confirmed CR or PR), disease control rate (DCR, defined as the proportion of patients with the best overall response of confirmed CR or PR, or SD), duration of response (DOR, defined as the time from the first CR or PR to the first documented disease progression or death of any cause), and safety.

### Statistical analysis

The phase 1b stage of the trial employed a standard 3 + 3 dose‐escalation design. Three patients at least and 18 patients at most (six for each dose) were included at this stage.

In the phase 2 stage, the estimated mPFS with platinum plus pemetrexed was 4.5 months,^8^, ^13^ and the estimated mPFS with the addition of anlotinib was 7.5 months.^5^, ^14^ The enrollment period and follow‐up duration were both 12 months. Therefore, 39 patients were needed with *α* of 0.05 and power of 80%. Considering a drop‐out rate of 10%, a total of 44 patients were needed at this stage.

All the statistical analyses were performed using SAS version 9.1.3 (SAS Institute). Continuous variables with normal distribution are presented as means ± standard deviations, and those that did not conform to normal distribution were presented as medians (ranges). Categorical data are described as numbers (percentages). PFS and DOR were estimated using the Kaplan–Meier method, and the corresponding 95% confidence intervals (CIs) were calculated using the Clopper‐Pearson method.

## RESULTS

### Patient characteristics

**FIGURE 1:**
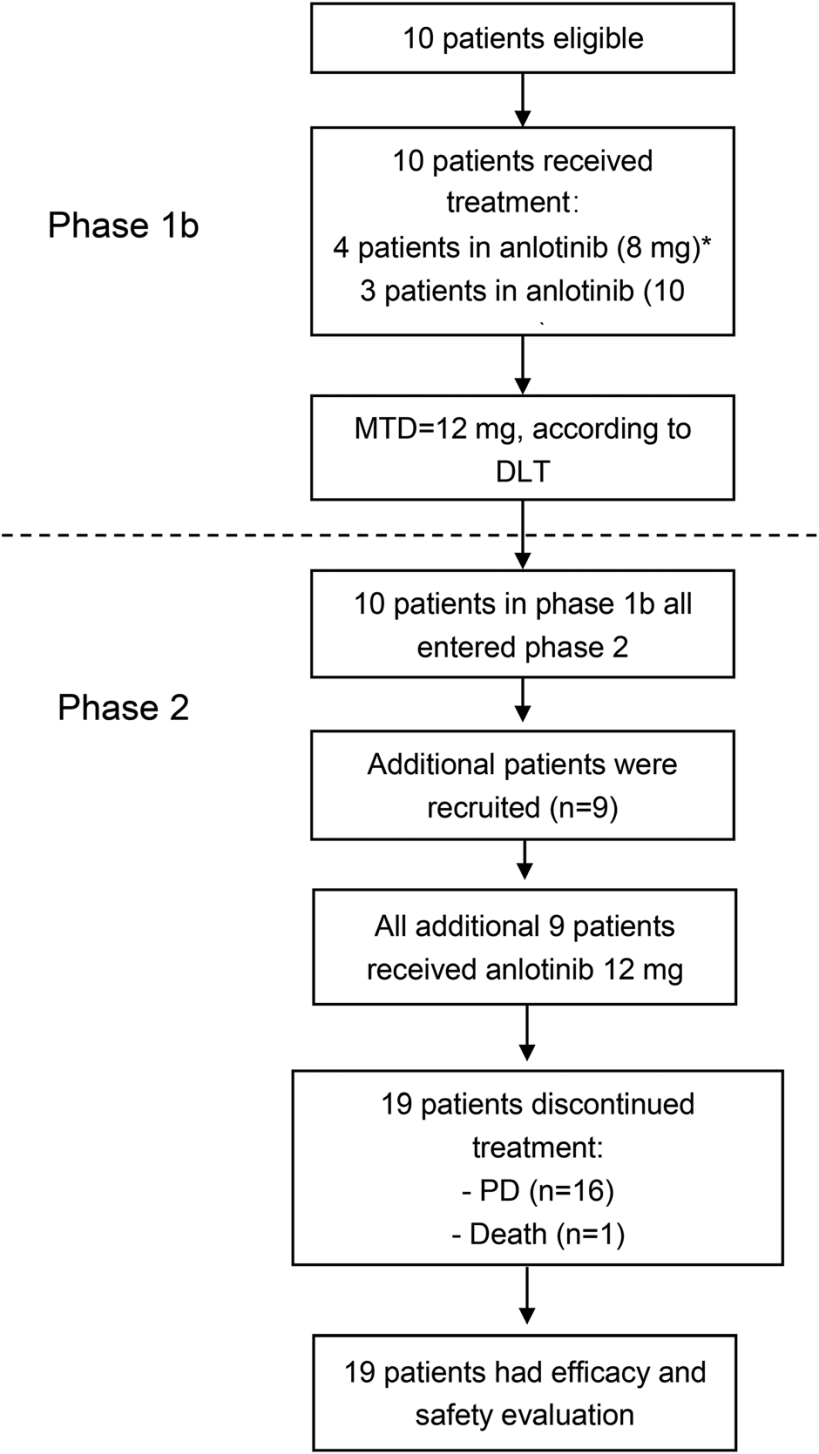
CONSORT diagram. *One patient in the 10 mg dose group was mistakenly administered 8 mg anlotinib, resulting in four patients in the 8 mg group. DLT, dose‐limiting toxicity; MTD, maximum tolerated dose; PD, progressive disease

The study was prematurely terminated in March 2021 because of slow accrual. Between January 18, 2019, and March 21, 2021, a total of 19 patients were enrolled in this study (Figure 1). One patient with a planned 10 mg dose was mistakenly administered with 8 mg anlotinib, resulting in four patients in the 8 mg group. All 10 patients in the phase 1b stage entered the phase 2 stage, and an additional nine patients were recruited in the phase 2 stage. The baseline patient characteristics are presented in Table 1. The median age was 61 (range, 49–70) years, and 13 (68.4%) patients were females. Brain metastases occurred in 10 (52.6%) patients. Patients previously received treatment with gefitinib (11 [58.0%]), icotinib (4 [21.0%]), and erlotinib (4 [21.0%]).

**TABLE 1:** Characteristics of all patients

Characteristics	Patients (*n* = 19)
Age (years), median (range)	61 (49–70)
Gender, *n* (%)	
Male	6 (31.6%)
Female	13 (68.4%)
Smoking, *n* (%)	
Current smoker	0
Previous smoker	5 (26.3%)
Never smoked	14 (73.7%)
ECOG performance status, *n* (%)	
0	6 (31.6%)
1	13 (68.4%)
Brain metastases, *n* (%)	
Yes	10 (52.6%)
No	9 (47.4%)
Tumor histology, *n* (%)	
Adenocarcinoma	19 (100%)
Others	0
Prior radiotherapy, *n* (%)	
Yes	8 (42.1%)
No	11 (57.9%)
First‐line TKI treatment, *n* (%)	
Gefitinib	11 (58.0%)
Icotinib	4 (21.0%)
Erlotinib	4 (21.0%)

Abbreviations: ECOG, Eastern Cooperative Oncology Group; TKI, tyrosine kinase inhibitor.

### Preliminary efficacy

In the phase 1b stage, none of the three patients in the 12 mg group had DLT. The MTD of anlotinib at 12 mg was adopted at the phase 2 stage.

**FIGURE 2:**
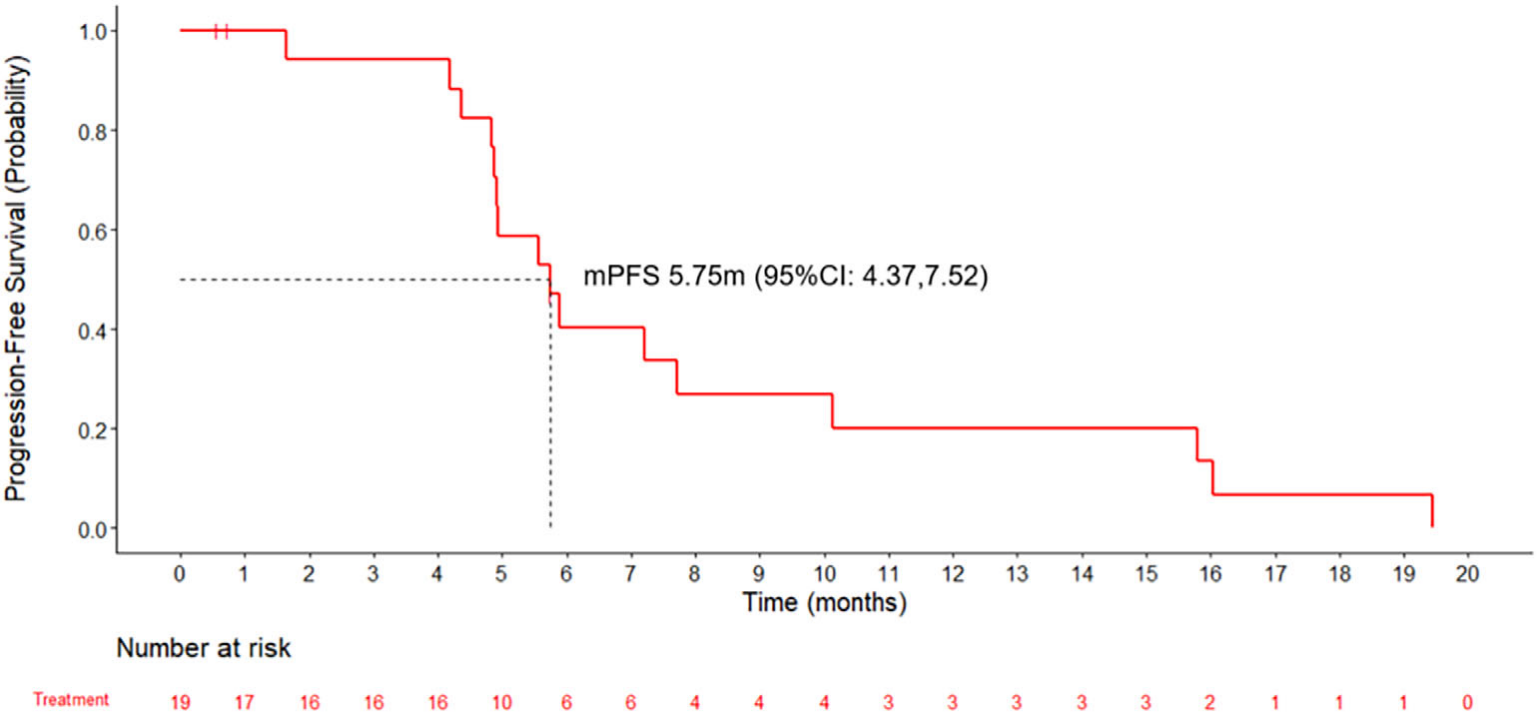
Progression‐free survival. mPFS, median progression‐free survival

As of the data cutoff date on August 11, 2021, the median follow‐up duration was 12.91 (range, 4.50–16.00) months. As shown in Figure 2, the mPFS was 5.75 (95% CI: 4.37–7.52) months.

**FIGURE 3:**
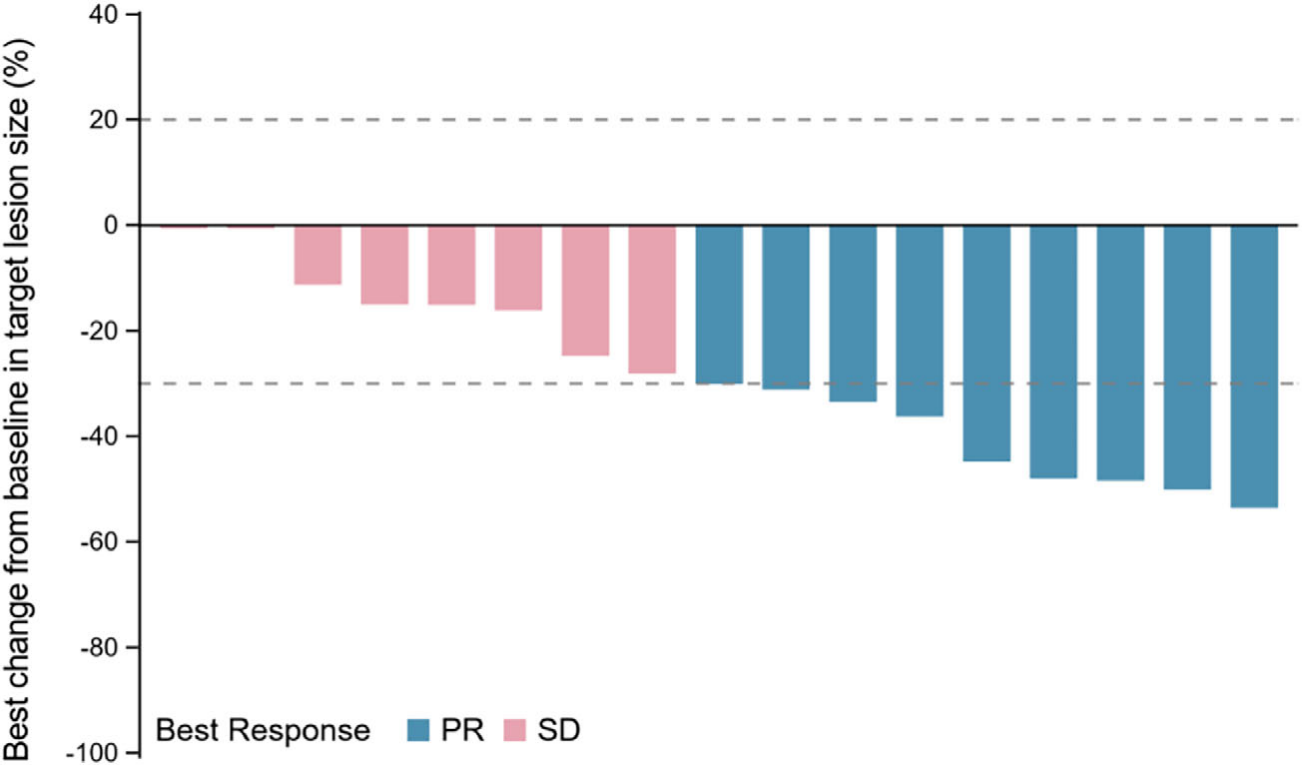
Waterfall plot of best change from baseline in tumor size (not evaluated for two patients). PR, partial response; SD, stable disease

**FIGURE 4:**
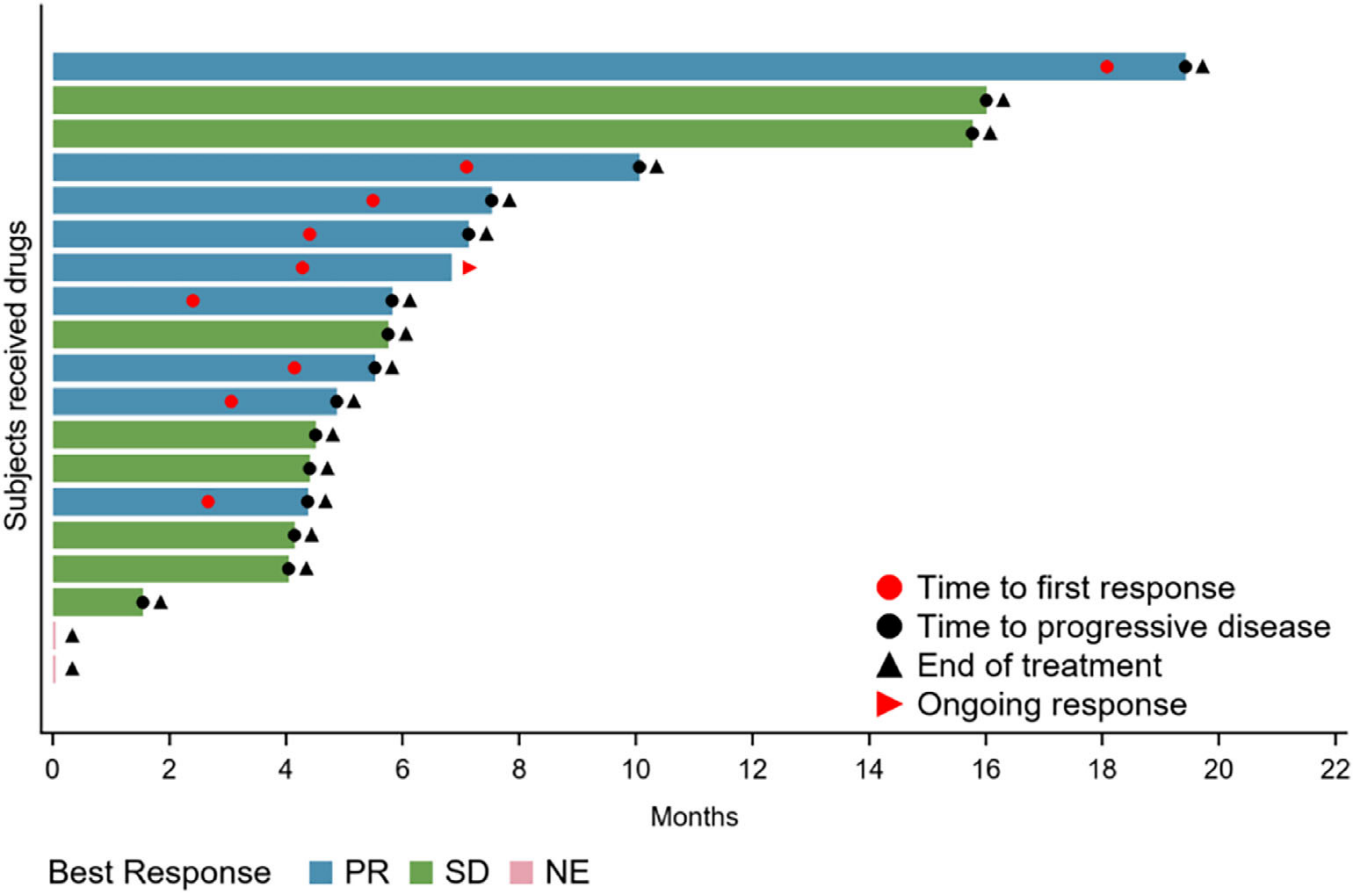
Swimming plot of duration of response in individual patients. NE, not evaluated; PR, partial response; SD, stable disease

Nine patients achieved PR, whereas eight patients had SD. The ORR was 47.4% (95% CI: 24.5%–71.1%) and DCR was 89.5% (95% CI: 66.9%–98.7%) (Table 2). The waterfall plot of the best change in tumor size from baseline for the individual patients is displayed in Figure 3. By the cutoff date, one patient with sustained PR was still on treatment, while the other 18 patients had discontinued treatment. The median DOR was 4.27 (95% CI: 2.4–7.1) months. The swimming plot of DOR in the individual patients is shown in Figure 4.

**TABLE 2:** Treatment response

Variable	Patients (*n* = 19)
Best overall response, *n* (%)	
Complete response	0
Partial response	9 (47.4%)
Stable disease	8 (42.1%)
Disease progression	0
Not evaluated	2 (10.5%)
Objective response rate (%)	47.4% (95% CI: 24.5%–71.1%)
Disease control rate (%)	89.5% (95% CI: 66.9%–98.7%)

Abbreviation: CI, confidence interval.

### Safety

Table 3 presents the treatment‐related AEs (TRAEs). In the 12 mg group, 11 (91.7%) and seven (58.3%) patients experienced any grade and grade 3–4 TRAEs, respectively. The most common TRAEs among the 12 patients in the 12 mg group were hypertension (8 [66.7%]), hypertriglyceridemia (8 [66.7%]), decreased platelet count (7 [58.3%]), hypercholesterolemia (6 [50.0%]), increased thyroid‐stimulating hormone (6 [50.0%]), increased ALT (5 [41.7%]), increased AST (4 [33.3%]), and proteinuria (4 [33.3%]). The most common grade ≥3 TRAEs were hypertension (6 [50.0%]), decreased platelet count (2 [16.7%]), and hypertriglyceridemia (1 [8.3%]).

**TABLE 3:** Any grade TRAE occurring in >30% of patients and all grade 3 or 4 TRAEs

Event	TRAE, *n* (%)			Grade 3–4 TRAE, *n* (%)		
	8 mg (*n* = 4)	10 mg (*n* = 3)	12 mg (*n* = 12)	8 mg (*n* = 4)	10 mg (*n* = 3)	12 mg (*n* = 12)
All	3 (75.0)	3 (100.0)	11 (91.7)	1 (25.0)	2 (66.7)	7 (58.3)
Hypertension	2 (50.0)	3 (100.0)	8 (66.7)	0	1 (33.3)	6 (50.0)
Hypertriglyceridemia	1 (25.0)	3 (100.0)	8 (66.7)	0	0	1 (8.3)
Platelet count decreased	1 (25.0)	1 (33.3)	7 (58.3)	0	0	2 (16.7)
Hypercholesterolemia	3 (75.0)	1 (33.3)	6 (50.0)	0	0	0
Thyroid‐stimulating hormone increased	2 (50.0)	2 (66.7)	6 (50.0)	0	0	0
Alanine aminotransferase increased	1 (25.0)	1 (33.3)	5 (41.7)	0	0	0
Aspartate transaminase increased	2 (50.0)	1 (33.3)	4 (33.3)	0	0	0
Proteinuria	1 (25.0)	1 (33.3)	4 (33.3)	0	0	0
Fatigue	2 (50.0)	2 (66.7)	2 (16.7)	0	0	0
Hand‐foot syndrome	2 (50.0)	1 (33.3)	2 (16.7)	0	0	0
Hyperuricemia	1 (25.0)	2 (66.7)	2 (16.7)	1 (25.0)	1 (33.3)	0
Numbness in extremities	1 (25.0)	2 (66.7)	0	0	0	0
Elevated lipase	1 (25.0)	1 (33.3)	0	0	1 (33.3)	0
Elevated D‐dimer	0	2 (66.7)	1 (8.3)	0	0	0
White blood cell decreased	0	1 (33.3)	1 (8.3)	0	0	0
Abdominal pain	0	1 (33.3)	1 (8.3)	0	0	0
Increased creatinine	0	1 (33.3)	1 (8.3)	0	0	0
Abnormal liver function	0	2 (66.7)	0	0	0	0
Increased γ‐glutamyl transpeptidase	0	1 (33.3)	0	0	0	0
Back pain	0	1 (33.3)	0	0	0	0
Hypoproteinemia	0	1 (33.3)	0	0	0	0
Abdominal distension	0	1 (33.3)	0	0	0	0
Anemia	0	1 (33.3)	0	0	0	0
Increased fibrinogen degradation products	0	1 (33.3)	0	0	0	0
Free triiodothyroxine decreased	0	1 (33.3)	0	0	0	0
Neutrophil count decreased	0	1 (33.3)	0	0	1 (33.3)	0

Abbreviation: TRAE, treatment‐related adverse event.

The dose of anlotinib was reduced in three (15.8%) patients. Three (15.8%) patients had AE that resulted in treatment interruption. No patients terminated treatment due to AEs, and no treatment‐related deaths occurred.

## DISCUSSION

This study investigated the activity and safety of anlotinib combined with platinum plus pemetrexed in patients with T790M‐negative *EGFR*‐mutant advanced non‐sqNSCLC after acquiring resistance to first‐ or second‐generation EGFR TKIs. This combination regimen showed promising activity with an mPFS of 5.75 months, an ORR of 47.4%, and a DCR of 89.5%. The toxicity was manageable.

This study was designed in 2018, and the efficacy and safety of immunotherapy in EGFR‐positive patients were controversial at that time. Immunotherapy combined with other therapies has been widely used in recent years. Immunotherapy agents have been increasingly used as second‐ or further‐line treatment, providing more treatment options for patients with advanced NSCLC. Evidence of immunotherapy‐based combination therapy in patients with advanced NSCLC was increasing. For instance, the IMpower150 trial showed that atezolizumab plus bevacizumab plus carboplatin plus paclitaxel could improve survival outcomes in patients with EGFR sensitizing mutations who had failed TKI therapy.^15^ Toripalimab plus pemetrexed and carboplatin could be another potential treatment option for these patients, as reported in the CT18 trial.^7^ As a result, patients often considered that immunotherapy plus chemotherapy with or without targeted therapy was more likely to achieve satisfactory efficacy, and they were less willing to accept the treatment regimen in this study (anlotinib plus chemotherapy). Therefore, it was difficult to recruit patients for our study, and the study was halted after enrollment of 19 patients.

The MTD of anlotinib was 12 mg once daily at a schedule of 14 days on followed by 7 days off, with no DLT in three patients in the phase 1b stage of this study. This treatment regimen was consistent with the dose recommendation of anlotinib monotherapy for advanced refractory solid tumors in a previous phase 1 study,^16^ indicating that there was no need to reduce the initial dose of anlotinib when combined with chemotherapy.

Treatments for patients with advanced NSCLC after acquiring resistance to first‐ or second‐generation EGFR TKIs are limited, especially for the T790M‐negative subset. Platinum‐based doublet chemotherapy with or without bevacizumab is the standard treatment for advanced NSCLC patients after failure of EGFR TKIs. The mPFS of platinum plus pemetrexed was 4.5 months, as mentioned above, and combining anlotinib with platinum and pemetrexed showed an improved mPFS of 5.75 months in this study. Bevacizumab plus platinum and pemetrexed were reported to achieve even better mPFS (7.4–10.8 months),^17^, ^18^ and the use of pemetrexed in the maintenance treatment might be an explanation. In this study, only anlotinib was used in the maintenance treatment. This study was designed in 2018 when anlotinib had just been approved in China, and the safety profile of anlotinib was not fully explored. In order to reduce toxicity and ensure the safety of the participants, and also considering the convenience of oral administration of anlotinib monotherapy, pemetrexed was not applied in the maintenance treatment. Thus, the expected mPFS of 7.5 months was not reached in this study. Nevertheless, anlotinib plus chemotherapy induced fewer TRAEs, especially grade 3–4 TRAEs, compared with bevacizumab plus chemotherapy.^18^

Rechallenging with first‐ or second‐generation EGFR TKI monotherapy showed modest efficacy in previous reports, with an mPFS of 3.5–4.2 months.^19^, ^20^, ^21^ Immunotherapy with nivolumab monotherapy was also tried in this population, but the survival benefit was worse with an mPFS of 2.1 months.^22^ The third‐generation EGFR TKI, osimertinib, and lazertinib, exhibited a potential clinical activity, with an mPFS of 5.1–5.4 months.^23^, ^24^ However, combination therapy may bring further survival benefits. Toripalimab plus carboplatin and pemetrexed achieved an mPFS of 7.0 months in the CT18 trial.^7^ The IMPRESS study reported an mPFS of 6.7 months with gefitinib plus cisplatin and pemetrexed in the T790M‐negative subgroup.^6^ Notably, the combination therapy brought better survival benefits than monotherapy with TKI or immune checkpoint inhibitor (ICI). The mPFS of 5.75 months with anlotinib plus platinum and pemetrexed in our study also supported the use of combination therapy. Although combined immunotherapy might be the preferred choice of second‐line treatment for T790M‐negative patients, this study provided an alternative option for those patients who might be unsuitable for taking immunotherapy. The ORR and DCR were also lower for monotherapy with TKI or ICI (ORR: 17%–37%; DCR: 47%–84%) than for combination therapy (ORR: 36.8%–50%; DCR: 87.5%–95.8%).^6^, ^7^, ^20^, ^22^, ^23^, ^24^ The combination of antiangiogenic agent and chemotherapy warrants further investigation for T790M‐negative *EGFR*‐mutant advanced non‐sqNSCLC.

The AE profile was consistent with previous studies of anlotinib and chemotherapy.^8^, ^13^, ^14^ The incidence of TRAEs was relatively high, but most of them were manageable using symptomatic treatments and dose adjustment. No patients terminated the treatment owing to AEs. All these results indicated that anlotinib plus chemotherapy was well‐tolerated. The addition of anlotinib to chemotherapy did not significantly increase the incidence or severity of AEs. The most common grade 3 or worse TRAE was hypertension (50.0%). No new safety concern was found in this study.

A large number of T790M‐negative patients with resistance to EGFR TKIs and failure to combined regimens of chemotherapy and immunotherapy usually had the only further treatment choice of single‐agent chemotherapy. A case report described the feasibility of anlotinib combining docetaxel, which indicated the potential of anlotinib combined with docetaxel might be worth exploring in such patients.

The current study had several limitations. First, the sample size was relatively small due to early termination. Therefore, the statistical power was probably insufficient to draw firm conclusions, and the small number of patients prompts caution when interpreting the results. Nevertheless, the data have value for the planning of future trials. Second, the OS data were not mature. Third, there was no control group. A large‐scale randomized controlled trial with longer follow‐up is warranted in the future.

In conclusion, anlotinib combined with platinum plus pemetrexed showed promising activity with manageable safety in patients with T790M‐negative *EGFR*‐mutant advanced non‐sqNSCLC after progression on first‐ or second‐generation EGFR TKIs. This combination therapy might be a potential alternative option as a second‐line treatment. Therefore, this anlotinib‐based combination strategy is worthy of further investigation.

## CONFLICT OF INTEREST

The authors declare that there are no conflicts of interest.

## Supporting information


**TABLE S1** List of the study centers participated in this study

## Data Availability

The data supporting the findings of this study are available from the corresponding author upon reasonable request.
