# Supplementary material for: Anlotinib plus chemotherapy for T790M‐negative EGFR ‐mutant non‐sqNSCLC resistant to TKIs: A multicenter phase 1b/2 trial
Source: Thorac Cancer. 2022 Nov 8;13(24):3496–503. doi: 10.1111/1759-7714.14713 (PMC9750808; doi:10.1111/1759-7714.14713)
Supplement: Supplementary file 1 — TABLE S1 List of the study centers participated in this study [file TCA-13-3496-s001.docx]

**Table S1.** List of the study centers participated in this study

| Number | Name of the centers | Number of participants |
| --- | --- | --- |
| 1 | Sichuan Cancer Hospital & Institute | 19 |
| 2 | Chengdu Seventh People’s Hospital | 0 |
| 3 | Nuclear Industry 416 Hospital | 0 |
| 4 | Chengdu Fifth People’s Hospital | 0 |
| Total |  | 19 |
